# The Carnot Cycle and Heat Engine Fundamentals and Applications II

**DOI:** 10.3390/e24020230

**Published:** 2022-02-02

**Authors:** Michel Feidt

**Affiliations:** Laboratory of Energetics, Theoretical and Applied Mechanics (LEMTA), URA CNRS 7563, University of Lorraine, 54518 Vandoeuvre-lès-Nancy, France; michel.feidt@univ-lorraine.fr

This editorial introduces the second Special Issue entitled “Carnot Cycle and Heat Engine Fundamentals and Applications II” https://www.mdpi.com/si/entropy/Carnot_Cycle_II (accessed on 29 January 2022).

The editorial of this Special Issue comes after the review process. Nine papers have been published between 26 February 2021 and 4 January 2022 due to the COVID-19 pandemic. These papers are listed hereafter in the inverse order of date of publication. Thanks to all the authors for the various viewpoints expressed that unveil fundamental and application aspects of the Carnot cycle and heat engines.

Authors are from Europe (four papers) and China (five papers). Each paper has been viewed by 400 to 1100 persons, except the last published one. Four papers have been presently cited 8 to 20 times.

Five papers address heat engines and Carnot configurations [1,2,3,4,5]. Papers by [2,4,5] concern, respectively, diesel engine, Lenoir, and Brayton cycles. The papers by [1,2] are related to Carnot engines. However, these five papers address real, irreversible cases. Three papers from Chinese authors [2,4,5] deal with finite-time thermodynamics (FTT). Papers by [2,5] use numerical methods such as genetic algorithm NASCA II (through LINMAP method, TOPSIS method, and Shannon entropy method) to optimize engines. The various objectives considered include power, power density, ecological function, and first law efficiency.

The paper that discusses the Lenoir cycle is from a more conventional point of view. It deals with the steady flow (such as Chambadal's original modeling). Objectives are power and first law efficiency. The corresponding allocation of heat transfer conductance is proposed, due to finite size constraints (i.e., the Utotal imposed).

In [2], the authors consider the optimization of an irreversible Carnot engine, comparing the FTT approach to the finite speed thermodynamics approach (FST). The direct method combined with the first law efficiency takes irreversibility into account (heat transfer gradients, pressure losses, and mechanical frictions). The main results include the following:

- Maximum energy efficiency differs from maximum power through different variable piston speed values;
- Results obtained through the FST method are different from those obtained from the Curzon–Ahlborn model (with time duration), due to the steady-state hypothesis.

Paper [1] concerns the modified Chambadal model of Carnot engines. It, too, addresses irreversibility but from a global point of view. This paper completes and improves the one proposed in the preceding Special Issue. A sequential optimization corresponding to various finite physical dimensions constraints is developed with the three objectives of energy, first law efficiency, and power. Two new concepts of entropic action are proposed and used—entropic action relative to production of entropy and entropic action relative to the transfer of entropy.

Papers by [6,7] extend the configuration from engines to reverse cycle machines including Stirling refrigerating machine [6] and Brayton refrigerating machine [7]. The paper by [7] combines, in fact, direct and inverse Brayton cycles, constituting more of a system, with regeneration purposes (regeneration before the inverse cycle). Constraints regarding pressure losses and size are considered.

The study by [6] is, in fact, related to the paper by [7], published in the preceding Special Issue: It discusses a finite physical dimension in a Stirling refrigerating machine according to Schmidt modeling. The paper uses entropy and exergy analysis. The most important irreversibility mechanisms are thermal ones and, more precisely, those due to regeneration.

Papers of [8,9] are specific but very interesting.

In [8], the authors discuss the chemical aspects of entropy and exergy analysis, including reconsideration of concepts and definitions relating the entropy–exergy relationship, with applications in industrial engineering and biotechnologies. The main objective is to evaluate the performance associated with all interactions between the system and the external environment. This is a crucial challenge today due to environmental concerns.

Paper by [9] is related to a very important and up-to-date subject—superconducting quantum circuits. It concerns a new approach mixing finite-time and quantum thermodynamics: quantum heat engine cycle. Closely linked to these fundamental aspects are corresponding applications for quantum computers.

To conclude, this second Special Issue confirms and improves the preceding one in terms of the following aspects:

- Systematic consideration of irreversibility (more than endo-reversibility);
- Two ways of optimization—namely, sequential (mainly analytical) and multiobjective (mainly numerical) approaches;
- Various objectives including energy, power, and first law efficiency for the most used approach;
- Various constraints; from a general point of view, the use of what we introduce as finite physical dimensions of optimal thermodynamics (FDOT) with finite constraints (see the book of the author of this editorial);
- The evolution of research from basic cycle to complex systems.

Perhaps these features could pave the way toward a third Special Issue, to expand and build upon concepts and approaches presented thus far.
